# Predicting Loss of Ambulation in Limb Girdle Muscular Dystrophy R9

**DOI:** 10.1002/acn3.70299

**Published:** 2026-01-04

**Authors:** Chandra L. Miller, Lauren N. Coffey, Shelley R. H. Mockler, Katie M. Laubscher, Carrie M. Stephan, M. Bridget Zimmerman, Katherine D. Mathews

**Affiliations:** ^1^ Department of Pediatrics University of Iowa Carver College of Medicine Iowa City Iowa USA; ^2^ University of Iowa Carver College of Medicine Iowa City Iowa USA; ^3^ Center for Disabilities and Development University of Iowa Health Care Stead Family Children's Hospital Iowa City Iowa USA; ^4^ Department of Biostatistics, College of Public Health University of Iowa Iowa City Iowa USA; ^5^ Department of Neurology University of Iowa Carver College of Medicine Iowa City Iowa USA

**Keywords:** dystroglycanopathy, FKRP, limb‐girdle muscular dystrophy, loss of ambulation

## Abstract

**Background:**

Limb girdle muscular dystrophy type R9 (LGMDR9) results from biallelic variants in *FKRP*. There is limited data to predict loss of ambulation (LOA) among those with LGMDR9.

**Methods:**

Participants in an ongoing dystroglycanopathy natural history study (NCT00313677) with *FKRP* variants who had achieved ambulation and were more than 3 years old were included (*n* = 97). LOA was defined as self‐reported full‐time wheelchair use, weakness preventing completion of the 10‐m walk‐run test (10MWT) or 10MWT time > 30 s. Interval‐censored time‐to‐event analysis was used to determine median age at LOA. Receiver operating characteristic curves were used to examine the ability of 10MWT and 4‐stair climb (4SC) times to predict LOA.

**Results:**

Of 97 participants, 55 (57%) were homozygous for the c.826C>A founder variant. Thirty‐one participants lost ambulation; 15 (49%) were homozygous for c.826C>A. Earliest age at LOA was 9 years (non‐homozygous for c.826C>A). Median age at LOA for the cohort was 46.0 years. Performances on 10MWT and 4SC were highly predictive of LOA within 3 years, with areas under the ROC curve of 0.89 (10MWT) and 0.87 (4SC) when genotype was included in analysis. Optimal cutoffs for predicting LOA within 3 years differed by genotype and had acceptable sensitivity and specificity.

**Discussion:**

LOA among those with LGMDR9 is strongly predicted by performance on 10MWT and 4SC. These results demonstrate the real‐world significance of standardized motor function tests used in LGMDR9 clinical trials and aid in anticipatory guidance.

## 1 Introduction

The dystroglycanopathies are a group of autosomal recessive muscular dystrophies characterized by hypoglycosylation of alpha dystroglycan (α‐DG), an integral component of the dystrophin‐glycoprotein complex (DGC) [1]. Through its high affinity binding to extracellular matrix proteins, such as laminin, glycosylated α‐DG functions to stabilize the sarcolemma [2, 3]. There are at least 20 genes known to be involved in the glycosylation of α‐DG [4]. Variants in these genes cause a range of clinical phenotypes from congenital onset weakness with multisystem involvement to adult onset limb girdle muscular dystrophy (LGMD) [5]. Variants in the Fukutin‐Related Protein (*FKRP*) gene represent one of the most frequent causes of dystroglycanopathy [6, 7]. *FKRP*‐associated dystroglycanopathy typically has an LGMD phenotype (LGMDR9) [5]. There are several known *FKRP* founder variants, the most common of which is c.826C>A [8]. Individuals homozygous for this variant tend to have a milder clinical course and later disease onset compared to those with other genotypes [9]. Currently there are no treatments proven to be effective for dystroglycanopathies aside from symptomatic management and treatment of complications, although clinical trials are underway (NCT05230459, NCT04800874).

The results of motor outcome measures in *FKRP*‐associated dystroglycanopathies have been previously reported; these focus on the rate of decline over time and have been consistent in showing generally slow progression, with variation by genotype [10, 11, 12]. Here we relate results of standardized motor outcome tests to loss of functional ambulation in participants with LGMDR9, information that is important to support the clinical significance of outcome measures used in clinical trials and for counseling patients.

## 2 Methods

All individuals with a known or suspected dystroglycanopathy were eligible to participate in a natural history study (NCT00313677) with annual follow up. This study received initial approval from an institutional review board in 2005 and has been enrolling since 2006. All participants gave written informed consent prior to the initiation of any study procedures. Medical history and general examination were completed at enrollment, updated annually, and supplemented by review of medical records. Age at onset was defined as onset of clinical weakness or first episode of severe transient weakness due to rhabdomyolysis and was based largely on participant recall. Standardized motor function tests were carried out by physical therapists trained in clinical trial outcome measures as previously described [10]. The 10‐m walk‐run test (10MWT) and four‐stair climb (4SC) were assessed as predictors of LOA, as these are readily available tests for which we have longitudinal data that are often included in clinical trials.

Through August 2025, 97 study enrollees with LGMDR9, who ever achieved ambulation, and were greater than 3 years old at time of evaluation were included. If a participant was enrolled in an interventional clinical trial, data collected while taking the study drug was excluded from analysis.

We defined loss of functional ambulation as either self‐reported age at full‐time wheelchair use, a timed 10MWT of greater than 30 s or an inability to complete a 10MWT due to disease‐related weakness, consistent with definitions used previously in Duchenne muscular dystrophy (DMD) [13, 14, 15].

Nonparametric survival analysis for interval‐censored data was used to estimate the cumulative probability of LOA (with 95% CI) followed by the generalized log rank test to compare LOA “survival” distribution between genotype groups and between male and female participants. In addition, interval censored proportional hazard regression was used to examine the effect of genotype, sex, and their interaction on LOA.

To assess performance on the 10MWT and 4SC as predictors of LOA within 3 years, logistic regression analysis fitted by the generalized estimating equation (GEE) method was used, with the area under the receiver‐operator characteristic curve (AUC of the ROC) for the logistic model obtained by k‐fold cross‐validation. For these analyses, each of the timed results for the motor function tests for all examinations with subsequent follow‐up of at least 3 years or greater was included, with each measure paired with the outcome of LOA occurring (Yes or No) within 3 years of the test. By using the GEE method for fitting the logistic model, we were able to account for the correlation of data points collected over time from the same participant. LOA predictive cutoff‐points for 10MWT and 4SC from the ROC curves were determined using the criteria of “minimum distance from the perfect marker.” All statistical analyses were performed using SAS version 9.4, SAS/STAT 15.2.

## 3 Results

Of the 97 participants included in analysis, 55 (57%) were homozygous for the c.826C>A *FKRP* founder variant. Thirty‐one (32%) have lost ambulation. Demographics are described in Table 1, together with the number of participants who met each definition for loss of ambulation. Duration of observation varied; 13 individuals had only one visit, while 24 individuals (25%) had data collected over a period of > 10 years. Median years of follow up are shown in Table 1.

**TABLE 1:** Demographics and ambulation status.

	Cohort	c.826C>A homozygote	All other genotypes
*n* (%)	97	55 (57%)	42 (43%)
Female (%)	49 (51%)	32 (58%)	17 (40%)
Age at last follow up, years median (IQR)	31.4 (17.7–44.7)	39.6 (22.7–47.4)	19.0 (13.9–32.0)
Age at symptom onset, years median (IQR)	8.5 (3–15.5)	11.5 (8–20)	3 (2–9)
Age at LOA, years median (95% CI)	46.0 (42.9, 60.7)	60.7 (42.9, 73.6)	32.0 (19.8, 45.6)
Age at LOA in years, range	9–74	23–64	9–74
Duration of observation, years			
Whole cohort^a^	4.9 (1.5–9.8)	5.6 (1.8–10.2)	4.9 (1.1–8.6)
Ambulatory at last visit	4.4 (1.5–8.8)	3.7 (1.0–9.4)	5.0 (2.0–6.3)
Lost ambulation by all criteria	31 (32%)	15 (27%)	16 (38%)
By self‐report	8	3	5
By 10MWT > 30s	12	9	3
By 10MWT U	11	3	8

Abbreviations: 10MWT, 10‐m walk test; IQR, interquartile range; U, unable to attempt test due to disease.

^a^ This is total duration of observation, which includes follow‐up after LOA for those that had loss of ambulation.

**FIGURE 1:**
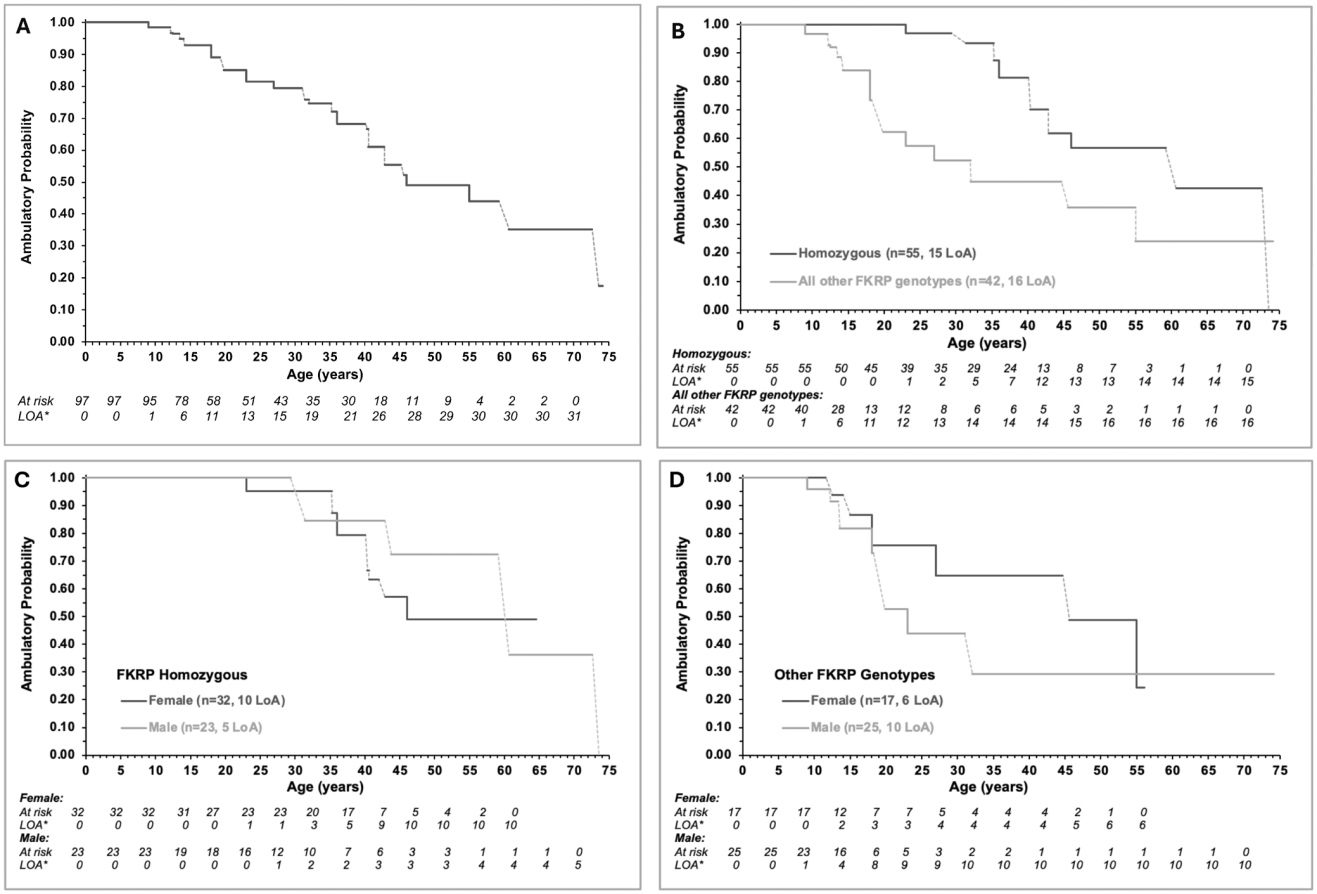
Survival analysis for loss of ambulation by age. (A) LOA for the entire cohort. (B) LOA by *FKRP* genotype. (C) LOA for those homozygous for *FKRP* variant c. 826C>A by sex. (D) LOA for those with other *FKRP* variants by sex. * for interval censored data where exact date of LOA is not known but occurred between a visit when LOA was noted and the prior visit where participant was still ambulatory, the midpoint was used to provide an estimate of the cumulative count for LOA at each specified timepoint.

The median age at LOA for the entire cohort is 46.0 years (95% CI: 42.9, 60.7), Figure 1A, Table 1. The median ages at LOA differed significantly by genotype (Figure 1B); median age at LOA for those homozygous for the FKRP c.826C>A variant was 60.7 years (95% CI: 42.9, 73.6), compared to 32.0 years (95% CI: 19.8, 45.6) for those with other genotypes (*p* < 0.0001). There was a wide range in age at loss of ambulation observed across the whole cohort, from LOA at age 9 years to ambulant at 74 years (Table 1).

There was no significant difference in age at loss of ambulation between males and females, for either genotype group (genotype‐sex interaction *p* = 0.26; Figure 1C,D). The hazard ratio for LOA, female vs. male, was 0.82 (95% CI: 0.40, 1.67) *p* = 0.58.

Disease duration from self‐reported age at first symptoms to age at loss of ambulation was significantly longer among those homozygous for the c.826 C>A mutation, [median disease duration at LOA of 36 years (95% CI: 33.2, 46.7) (range 12–60 years)], compared to those with other mutations [median disease duration at LOA was 20 years (95% CI: 17.0, 25.0) (range 4–40 years)] (*p* = 0.002).

The logistic regression analysis for LOA within 3 years with 10MWT as predictor used data from 59 (32 homozygous for the FKRP c.826C>A variant and 27 with other genotype) of the 97 participants. Twelve participants who had LOA prior to initial visit and 26 with less than 3 years follow‐up were excluded from the analysis. There were 4 additional participants who were not able to do 4SC at initial visit due to disease that were excluded in the 4SC analysis. There was no significant difference between those included in the logistic regression analysis and those excluded due to short follow‐up in age at onset (*p* = 0.73), age at initial visit (*p* = 0.97), and initial 10MWT speed (*p* = 0.52).

**FIGURE 2:**
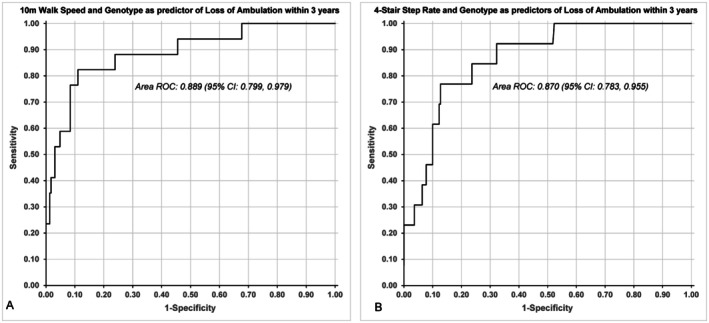
Prediction of loss of ambulation within 3 years including genotype as a predicator by ROC analysis. (A) Loss of ambulation by 10 m walk time. (B) Loss of ambulation by 4SC time.

Logistic regression analysis for LOA within 3 years, with genotype as covariate, showed a significant association with 10MWT and with 4SC (both *p* < 0.0001). Both motor tests were highly predictive of LOA within 3 years with area under the ROC curve (AUC) for 10MWT of 0.889 (95% CI: 0.799, 0.979) and 0.870 (95% CI: 0.783, 0.955) for 4SC (Figure 2). The predictive cut points for 10MWT and 4SC by genotype for the outcome of LOA within 3 years determined from the ROC curves are shown in Table 2.

**TABLE 2:** 10 m walk or 4‐stair climb, and *FKRP* genotype as predictor of loss of ambulation within 3 years.

	10 m walk	4‐stair climb^a^
Number of cases (measurements)	59 (243)	55 (233)
LOA within 3 years	17	13
AUC of ROC (95% CI)	0.889 (0.799, 0.979)	0.870 (0.783, 0.955)
Prediction cut‐off		
Homozygous	13.22 s	10.81 s
All other genotypes	8.27 s	5.17 s
Sensitivity	82.4%	76.9%
Specificity	88.9%	87.3%

^a^ Among those able to do 4‐stair climb.

## 4 Discussion

Loss of ambulation is an important milestone in disease progression for people with LGMDR9. Using prospectively collected longitudinal data on a large LGMDR9 cohort, we demonstrate that performance on standardized motor outcome measures is strongly predictive of loss of ambulation within 3 years across *FKRP* genotypes, confirming the importance of these measures as outcomes of clinical importance in trials. Our predictive cut‐point values for 10MWT and 4SC can be useful in clinical discussions around future loss of ambulation.

There is limited information on predicting LOA in people with LGMDR9. A previous analysis of individuals with LGMDR9 found that baseline distance walked in 6 min (6MWT) was not clearly predictive of loss of ambulation within 6 years [12], however, this study was limited to those homozygous for the c.826C>A variant and only six individuals lost ambulation during the period of observation. We did not include 6MWT performance in the current analysis, but previously showed that it is highly correlated with 10MWT [10]. We note that the period of observation for our cohort extended well beyond 6 years, which is required to fully understand the natural history of a slowly progressive disease. We show that 10MWT and 4SC times can be used to predict LOA within 3 years with sensitivity and specificity of > 80% for 10 MWT and slightly lower sensitivity for 4SC, information that can be used in anticipatory guidance in the clinic. Similar methodology was used to predict LOA for those with Duchenne muscular dystrophy, a more rapidly progressive and homogeneous form of muscular dystrophy. In DMD, timed function tests (4SC, 10MWTand time to stand) predicted LOA in 1 year with high sensitivity and specificity [16].

We show a significant effect of *FKRP* genotype on LOA, consistent with previous reports, and a wide range in variability even within genotypes. Various methodologies have been used to report LOA. A recent systematic review of the literature reported LOA for those with adult‐onset LGMDR9 at a mean age of 49.4 (SD: 14.4) years, mid‐childhood onset at 21.7 (SD: 9.4), and early childhood onset at 16.9 (SD: 11.1) years [17]. A large Norwegian cohort found age at LOA for people with LGDMR9 who are homozygous for the c. 826C>A variant (87.6% of the cohort) had a mean age at LOA of 36.5 years, much younger than the median of 60.7 years (95% CI: 42.9, 73.6) observed in our series [18, 19]. For those with other genotypes, published studies report a mean LOA age of 13 and 20 years [18, 19], again younger than our median of 32.0 years (95% CI: 19.8, 45.6). We note broad confidence intervals or standard deviations in all large studies, including the present one, suggesting factors contributing to individual variation in motor function outside of simple *FKRP* genotype. Our numbers are closer to those in the systematic literature review but our cohort is generally older at LOA than previous reports. Possible explanations for the observed variations in LOA between cohorts include relatively small sample sizes (particular among rarer genotypes), regional differences in clinical practice around wheelchairs, methodologic differences and background genetic factors. In addition, as our cohort was seen at a single center and many participants travel to attend study visits, we might have participation bias favoring more mildly affected individuals, although the wide range in age at LOA suggests we include a full range in severity.

In contrast to the differences reported in average age at LOA, the youngest ages at LOA were nearly identical in our series compared to the Norwegian cohort [18]. In our series, the earliest age at loss of ambulation was 23 years for a participant homozygous for c. 826C>A and 9 years for other genotypes, while the earliest ages at LOA were 30 and 9 years respectively in the Norwegian cohort [18]. Considering time from symptom onset to age at LOA, we found a median disease duration of 36 years for those homozygous for c.826 C>A and 19 years for those with other genotypes, 8–9 years longer compared to the Norwegian series [18].

Published LGMDR9 registry (*n* = 305) data includes current age of those who use a wheelchair rather than age at LOA. Among those homozygous for c. 826C>A, 18% use a wheelchair full time at a mean age of 53.2 ± 12.7 years compared to 40% of the registry respondents with other mutations at a mean age of 32.6 ± 12.0 years [20]. Consistent with this registry data, the majority of our cohort retained ambulation into adulthood. Importantly, both our homozygous and non‐homozygous cohorts included ambulatory participants over the age of 60.

An effect of sex on rate of LGMDR9 progression was suggested in the Norwegian cohort, with males progressing more rapidly than females [18]. In contrast, LGMDR9 registry data suggest that for most ages, females homozygous for c. 826C>A were more likely to use a wheelchair than were males, while there was no clear difference for those with other mutations [20]. LOA did not differ by sex in the present study.

Loss of ambulation has been studied in other forms of recessive LGMD, including sarcoglycanopathies (*n* = 70), calpainopathy (*n* = 111), and dysferlinopathy (*n* = 109) where the median ages of full‐time wheelchair use were 18, 45, and 56 years, respectively [21]. A study specifically examining phenotypic differences between subtypes of sarcoglycanopathies found that 56.3% of alpha‐ (*n* = 159), 78.4% of beta‐ (*n* = 73), and 84.4% of gamma‐sarcoglycanopathy patients (*n* = 157) lost ambulation by 18 years old [22]. Together with LGMDR9 data, these studies demonstrate the wide variation across recessive LGMDs and specific LGMD genotypes in progression to the important disease milestone of full‐time wheelchair use.

Our cohort includes a wide range of participant ages and genotypes. A limitation of our study is the relatively small number of participants who lost ambulation. This is not unexpected in a rare and often slowly progressive disease. As noted above, we might have selected for individuals able to travel, favoring the milder end of the phenotypic spectrum. We chose to focus on 10 MWT and 4 SC as these are commonly used outcomes in LGMD studies and have been followed for up to 20 years in our cohort. In previous work, we demonstrated high correlation among motor outcome measures [10]. We lack sufficient follow up with newer outcome measures such as the North Star Assessment for Limb‐Girdle Type Dystrophies (NSAD) to associate performance with LOA [10].

The clinical progression of *FKRP*‐related muscular dystrophy is highly variable and strongly influenced by genotype. Loss of ambulation is an important clinical landmark in disease course for people with LGMD R9 and our results can aid in anticipatory guidance, although the broad variation even within genotypes must also be discussed. These data also demonstrate the real‐world significance of standard motor function tests in predicting disease progression. As new treatments are developed, we expect that this natural history will be modified.

## Author Contributions

Conceptualization and design: K.D.M., C.L.M., L.N.C. Funding acquisition and project supervision: K.D.M. Project administration: C.M.S., K.D.M. Data acquisition: S.R.H.M., K.M.L., C.M.S., C.L.M., K.D.M. Methodology and data analysis: M.B.Z. Original draft preparation: L.N.C., K.D.M., C.L.M. Preparation of figures and tables: M.B.Z., K.D.M., C.L.M. Review and editing: all authors.

## Funding

This work was supported by the National Institutes of Health, NIH U54 NS053672.

## Conflicts of Interest

Chandra L. Miller, DNP, receives funding from the Paul D. Wellstone Muscular Dystrophy Cooperative Research Center grant (NIH U54 NS053672), and clinical trial or registry funding from AMO, AskBio, AveXis, Avidity, Biohaven, Capricor, Cure Duchenne, Edgewise, Genethon, GRASP, Italfarmaco, Larimar, MDA, ML Bio, Pfizer, PPMD, PTC Therapeutics, RegenxBio, Sarepta Therapeutics, and Scholar Rock.

Lauren N. Coffey, BS, received funding from the University of Iowa Carver College of Medicine and the Paul D. Wellstone Muscular Dystrophy Cooperative Research Center grant (NIH U54 NS053672).

Shelley R. H. Mockler, DSc, receives funding from NIH grant 2 U54 NS053672‐11, and Italfarmaco, ML Bio Solutions, Edgewise Therapeutics, Biohaven, Capricor Therapeutics Inc., Sarepta Therapeutics, Ask Bio, Pfizer Inc., Scholar Rock, Avidity Biosciences, and Shire/Takeda Pharmaceutical. She serves as a paid consultant for Avidity Biosciences and ATOM International.

Katie M. Laubscher, DPT, receives funding from NIH grant 2 U54 NS053672‐11, Edgewise Therapeutics, ML Bio Solutions, Ask Bio, Capricor Therapeutics Inc., Scholar Rock, Avidity Biosciences, Biohaven, Italfarmaco, and Sarepta Therapeutics Inc. She serves as a paid consultant for ATOM International.

Carrie M. Stephan, MA, receives funding from NIH grant 2 U54 NS053672‐11, and Sarepta Therapeutics Inc.

Dr. M. Bridget Zimmerman receives funding from NIH grants 5 P50 NS053672, 1 R01 NR017610‐01A1, 4 UH3 AR076387‐02, 5 R01 CA246540‐05, 5 R01 HL153095‐04, 5 R01 AR077418‐04, 1 R25 HL161716‐02, 5 R01 HD104616‐03, 5 R01 HL164662‐02, 1 R56 AG080101‐01A1, and 1 R01 HL173483‐01.

Katherine D. Mathews receives research funding from the Paul D. Wellstone Muscular Dystrophy Cooperative (NIH U54 NS053672) and the Centers for Disease Control (U01 DD001248). She serves as an advisory board member for MDA and the FSH Society; is a board member for the Friedreich Ataxia Research Alliance (FARA); receives clinical trial funding from PTC Therapeutics, Sarepta Therapeutics, Pfizer, Italfarmaco, Biohaven, AMO, Capricor, AskBio, ML Bio, Scholar Rock, Avidity, Edgewise; serves as an industry consultant for Sarepta, AskBio, ML Bio, and Edgewise; and serves on safety review boards for Sarepta and Dyne.

## Data Availability

The data that support the findings of this study are available in the main body of this article. Additional data that are not publicly available due to privacy or ethical restrictions are available on request from the corresponding author.
